# Identifying Key Challenges Facing Healthcare Systems In Africa And Potential Solutions

**DOI:** 10.2147/IJGM.S223882

**Published:** 2019-11-06

**Authors:** Obinna O Oleribe, Jenny Momoh, Benjamin SC Uzochukwu, Francisco Mbofana, Akin Adebiyi, Thomas Barbera, Roger Williams, Simon D Taylor-Robinson

**Affiliations:** 1 Excellence and Friends Management Care Centre (EFMC), Abuja, Nigeria; 2 Department of Epidemiology and Community Medicine, Federal University Lafia, Nasarawa State, Nigeria; 3 Department of Community Medicine, College of Medicine, University of Nigeria, Enugu, Nigeria; 4 National AIDS Commission, Maputo, Mozambique; 5 University College Hospital, Ibadan, Nigeria; 6 Liver Unit, Department of Surgery and Cancer, St Mary’s Campus, Imperial College London, London W2 1PG, UK; 7 Institute of Hepatology, London SE5 9NT, UK

**Keywords:** Africa, healthcare management, human resources, health systems

## Abstract

**Introduction:**

Healthcare systems in Africa suffer from neglect and underfunding, leading to severe challenges across the six World Health Organization (WHO) pillars of healthcare delivery. We conducted this study to identify the principal challenges in the health sector in Africa and their solutions for evidence-based decisions, policy development and program prioritization.

**Methods:**

The study was conducted as part of a recent African Epidemiological Association Meeting in Maputo, Mozambique with participants drawn from 11 African countries, Cuba, Portugal and the United Kingdom. Participants were divided into 10 groups, consisting of 7 to 10 persons each. Brainstorming approaches were used in a structured, modified nominal group process exercise to identify key challenges and strategies to mitigate healthcare service challenges in Africa. Identified challenges and solutions were prioritised by ranking 1–5, with 1 most important and 5 being least important.

**Results:**

The first three challenges identified were inadequate human resources (34.29%), inadequate budgetary allocation to health (30%) and poor leadership and management (8.45%). The leading solutions suggested included training and capacity building for health workers (29.69%), increase budgetary allocation to health (20.31%) and advocacy for political support and commitment (12.31%).

**Conclusion:**

The underdeveloped healthcare systems in Africa need radical solutions with innovative thought to break the current impasse in service delivery. For example, public-private initiatives should be sought, where multinational companies extracting resources from Africa might be encouraged to plough some of the profits back into healthcare for the communities providing the workforce for their commercial activities. Most problems and their solutions lie within human resources, budget allocation and management. These should be accorded the highest priority for better health outcomes.

## Introduction

Globally, countries are faced with health system problems which vary from one to the next. While health service delivery challenges are more often seen in countries with a very high Human Development Index (HDI), human resources challenges attract more attention within those with a low HDI.^1^ Healthcare systems in Africa have, over the years, suffered from man-made issues which cut across institutional, human resources, financial, technical and political developments. With this in mind, the World Health Organization (WHO) in 2007 proposed a framework that describes healthcare systems in terms of six core components or “building blocks”: i) service delivery; ii) healthcare workforce; iii) healthcare information systems; iv) medicines and technologies; v) financing; and vi) leadership/governance.^2^ The majority of African countries are unable to meet the basic requirement for good healthcare systems. Poor governance and human resource challenges are linked to ineffective integration of services in resource-limited nations.^3^–^5^

Dilapidated healthcare systems have facilitated medical tourism, for example, leading to over 5000 people leaving Nigeria every month for various forms of treatment abroad and about 1.2 billion US dollars lost from the Nigerian economy to medical tourism yearly.^6^ Other healthcare system problems prevalent in Africa include financial barriers to healthcare services with high rates of out-of-pocket expenditure, owing to ineffective national health insurance systems,^7^–^9^ and poor service integration.^3^,^8^ Human resources shortages and “brain drainage” from Africa to Europe, the Middle East and North America compound healthcare outcomes.^9^–^11^ Healthcare sector industrial action (healthcare worker strikes) is frequent in Nigeria, where it has complicated most aspects of the healthcare service delivery^10^–^14^ and consequently prevented optimal healthcare delivery to the Nigerian population.^15^

As part of efforts to remedy the lack of financial risk protection mechanisms in Africa, some countries, such as Nigeria, Ghana, Tanzania, Kenya, Rwanda and Ethiopia have started implementing social health insurance schemes.^7^–^9^ However, the majority of people still suffer financial barriers as out-of-pocket expenditure is required before medical care can be provided, even in emergency situations and importantly, many insurance programs leave out the poor.^16^ The poor therefore bear the highest burden of diseases and experience high levels of financially crippling healthcare expenditure in many sub-Saharan African countries.^16^,^17^ In countries like Nigeria, non-implementation of relevant policies, programs and agreements between government and the various healthcare workers have frequently led to employee industrial action and periodic refusal to provide healthcare services to the sick.^10^,^11^

Several studies have documented key healthcare system challenges globally with recommended solutions. However, challenges that healthcare systems in Africa face require in-depth exploration to identify, generate and implement contextual solutions that make significant population-level health gains with efficient use of resources.^18^ In order to bridge this information gap, the authors used the opportunity of a gathering of key opinion leaders at a recent African Epidemiological Association Annual Meeting in Maputo (with participants drawn from various countries in Africa) to identify perceived unmet needs in African healthcare systems and suggest ways of addressing them. This was done to determine healthcare system challenges which were most worrisome to principal African policy makers and implementers across Africa, as well as to identify major ways these challenges could be effectively addressed.

## Methods

The study was conducted as part of the African Epidemiological Association (AfEA) Annual Scientific and General Meeting in Maputo, Mozambique, between April 14 and 17th, 2019.

The conference, with the theme, “Epidemiology for Sustainable Development in Africa” had epidemiology, policy development and community health; data and the science of evidence; disease surveillance and response; environmental epidemiology; epidemiological transition; new tools and horizons in epidemiology; and delivery science and implementation research as subthemes. Participants focused on how to improve epidemiology practice and policies in Africa in particular, and the world at large. Plenary discussions, group work and scientific sessions were used to fully explore the various topics and a communiqué addressing the participants’ submissions, fears and solutions were delivered at the end of the conference.

A total of 77 participants, composed of programme implementing partners (n=40), policy-makers (n=5) and health researchers (n=13) were drawn from 11 African countries, Cuba, Portugal and the United Kingdom. Some participants were identified as belonging to multiple categories, either as both implementing partner and researcher (n = 24), researcher and policy-maker (n = 5), implementing partner and policy-maker (n = 1), or implementing partner, researcher and policy-maker (n = 6). The participants were divided into 10 groups with each group consisting of 7–10 persons. A brainstorming approach in a structured-modified nominal group process (NGP) exercise was used.^19^,^20^ This was conducted to identify key challenges facing healthcare services and develop strategies to mitigate the identified healthcare system challenges in Africa. Identified challenges and solutions were prioritised by ranking 1–5, with 1 being the most important and 5 being the least important. Participants included policy-makers, program managers and implementers, faculty of universities, executives of non-governmental organizations, researchers and residents in public health.

The NGP was conducted in two phases designed to maximise participant focus and engagement. During the first phase, each group member identified key challenges facing healthcare services and strategies to mitigate them in the African context. The groups then discussed and elaborated on these issues in the next NGP phase. Each of these sessions included three distinct activities, namely 1) generating key challenges, 2) generating key strategies/solutions, and 3) ranking the challenges and strategies.

We used “brainstorming” first as it is an effective and low-cost method of identifying key challenges and possible solutions to identified challenges.^21^ Individuals and groups identified between 3 and 7 challenges and solutions; generating a total of 275 challenges and 214 potential solutions. A team member was identified to document and present the findings to the meeting organizers.

The principal investigator, a public health physician, recruited 2 research assistants who acted as the facilitators of this study. The facilitators created the 10 groups, shared the study instrument and timed the whole exercise. Approval for the study was obtained from the AfEA Council and Executive and each participant provided consent to participate in the study.

### Data Analysis

We delinked data, entered findings into SPSS v23 (IBM Corp, Armonk, NY, USA) and MS Excel Sheet (Microsoft Inc., Redmond, WA, USA), analysed, and reported aggregated results. Four questionnaires were completed in Portuguese. These were translated and analysed by one of the authors who speaks Portuguese. Two of the authors analysed the results and developed themes base on similar entries. Results were further summarized along the line of the WHO health system pillars (building blocks)^1^ The most appropriate theme or block for each entry was used based on specialist (A Public health practitioner with post-graduate degree in public health) opinions. Results were presented in tables and charts.

## Results

Respondents came from 11 African countries, Cuba and Portugal with close to half of the respondents from Nigeria (44.2%), followed by Mozambique (31.2%), which was the host country for the conference. The majority of the respondents had an MPH or MSc (36.4%); this was followed by those who had a Medical College Fellowship (23.4%) or a PhD (14.3%). Over a half of the respondents were programme officers (54.6%), while about one-fifth of the respondents were academics (Table 1).

**Table 1:** Sociodemographic Characteristics Of Respondents

Variable	Frequency (N=77)	Percentage
**Countries**		
Nigeria	34	44.2
Mozambique	24	31.2
Botswana	4	5.2
Cameroon	2	2.6
Cuba	1	1.3
Liberia	1	1.3
Malawi	1	1.3
Mali	2	2.6
Portugal	1	1.3
Rwanda	1	1.3
Sierra Leone	2	2.6
South Africa	3	3.9
Uganda	1	1.3
**Highest Qualifications**		
Fellowship	18	23.4
PhD	11	14.3
MWACP^a^	4	5.2
MPH/MSc	28	36.4
MPhil	1	1.3
MBBS	9	11.7
BSC	3	3.9
Missing	3	3.9
**Ranks**		
Academics	15	19.5
Programme officers	42	54.6
Students	9	11.7
Resident doctors	7	9.1
Others	4	5.2

**Note:** ^a^Member West African College of Physicians.

The top four problems of the health sector in African countries identified by individual participants were inadequate human resources (n = 49 respondents, 17.82%), poor resource allocation to health (n = 48, 17.45%), poor maintenance of healthcare system infrastructure (n = 28, 10.18%) and lack of political will (n = 20, 7.27%). The rest includes lack of access to healthcare, weak healthcare systems, high disease burden, healthcare system corruption, poor leadership and administration, non-use of evidence-based intervention, poor quality of healthcare services, lack of good resource management, weak training and education of healthcare workers, weak healthcare management information systems (HMIS), non-prioritization of healthcare activities, and professional rivalry, as shown in Table 2.

**Table 2:** Challenges Facing Healthcare Services As Identified By Individuals

Challenges	Frequency (N=275)	Percentage
Inadequate human resources	49	17.82%
Poor resource allocation to health	48	17.45%
Poor maintenance of healthcare infrastructure	28	10.18%
Lack of political will	20	7.27%
Lack of access to healthcare	15	5.45%
Weak health systems	11	4.00%
High disease burden	10	3.64%
Health system corruption	10	3.64%
Poor leadership and administration	10	3.64%
Non-use of evidence-based intervention	8	2.91%
Poor quality of healthcare services	8	2.91%
Lack of good resource management	7	2.55%
Weak training and education	7	2.55%
Weak Health information Management System (HMIS)	6	2.18%
Non-prioritization of health activities	5	1.82%
Professional rivalry	5	1.82%
Poor motivation of health workers	4	1.45%
Poor integration of programmes	3	1.09%
Lack of community participation	3	1.09%
Poor technological advancement	2	0.73%
Unequal distribution of healthcare facilities	2	0.73%
Lack of medicines	2	0.73%
Low healthcare seeking behaviour	2	0.73%
Poor attitude of healthcare workers	2	0.73%
Poverty	2	0.73%
Lack of accountability and transparency	1	0.36%
Uncoordinated research at national level	1	0.36%
Poor regulatory capacity	1	0.36%
Migration of healthcare workers	1	0.36%
Incessant industrial actions	1	0.36%
Poor supervision	1	0.36%

According to the groups, the top four challenges were inadequate budgetary allocation (n = 10 groups, 21.7%), inadequate human resources for health (n = 9, 19.6%), ineffective leadership and management (n = 5, 10.9%), poor data quality with poor use of data for audit (n= 4, 8.7%) and lack of political will (n = 4, 8.7%). The rest included limited access to healthcare, inadequate healthcare infrastructure, weak healthcare systems, poor integration of healthcare programmes and professional rivalry.

**Figure 1:**
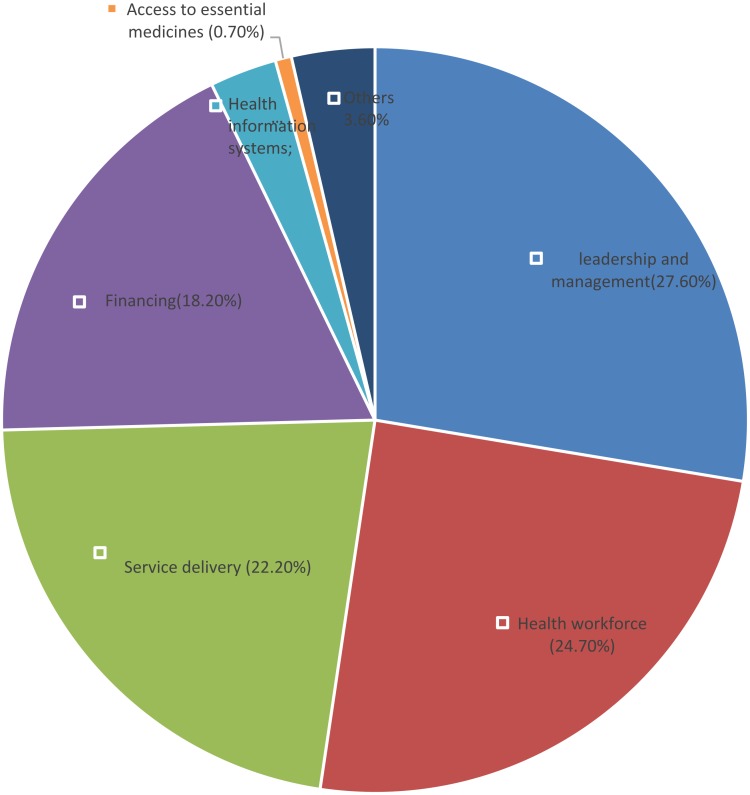
Challenges regrouped according to the six pillars of the health systems.

When these problems were regrouped, based on the six building blocks of the health system proposed by WHO, the leading problems clustered around leadership and governance (26.18%), healthcare workforce (25.45%) and healthcare service delivery (22.91%) (Figure 1).

Participants also individually and collectively identified some solutions to these challenges. The leading solutions suggested to the outlined challenges in the healthcare sector by individuals included training and capacity building of healthcare workers (n=43 respondents, 20.1%), adequate health insurance schemes (n = 20, 9.3%), advocacy and increase political engagement (n = 19, 8.9%), increased budgetary allocation to healthcare (n = 19, 8.9%) and improved healthcare infrastructure (n = 17, 7.9%). Other factors included increase collaboration among stakeholders to address healthcare issues, improved capacity to prevent and control disease, leadership and management training, improved monitoring and evaluation of healthcare services and better remuneration of healthcare workers, as shown in Table 3.

**Table 3:** Key Solutions Identified By Participants

Key Solutions	Frequency (N=215)	Percentage
Training and capacity building	43	20.1%
Health insurance	20	9.3%
Advocacy and increase political engagement	19	8.9%
Increase budgetary allocation to health	19	8.9%
Improve healthcare infrastructure	17	7.9%
Increase collaboration among stakeholders to address health issues	11	5.1%
Improved capacity to prevent and control diseases	8	3.7%
Leadership and management training	8	3.7%
Monitoring and evaluation of healthcare services	8	3.7%
Better remuneration of health workers	8	3.7%
Recruitment of healthcare workers	6	2.8%
Health system reforms and research	5	2.3%
Enhance implementation research	5	2.3%
Increase investment in most impactful interventions	5	2.3%
Research collaboration between institutions and ministries of health	4	1.9%
Accountability	4	1.9%
Integration of vertical programmes	4	1.9%
Prioritization of health intervention	3	1.4%
Use of expert managers	3	1.4%
Increase healthcare facilities	3	1.4%
Upgrading and equipping training institutions	2	0.9%
Strengthen health management information system	2	0.9%
Use of technology	1	0.5%
Revising training curriculum	1	0.5%
Strengthen regulatory capacity	1	0.5%
Mental and academic decolonization	1	0.5%
Poverty alleviation	1	0.5%
Community mobilization	1	0.5%
Resource mobilization	1	0.5%
	214	100.0%

Groups identified investment in capacity building/training of healthcare workers (n = 8 groups, 16.7%), advocacy for political support (n = 7, 14.6%), increased budgetary allocation (n = 6, 12.5%), and adequate health insurance schemes (n = 5, 10.4%). The rest included improved data management and use of data for action, upgrading of healthcare infrastructure, improve leadership and management and active recruitment of healthcare workers.

**Figure 2:**
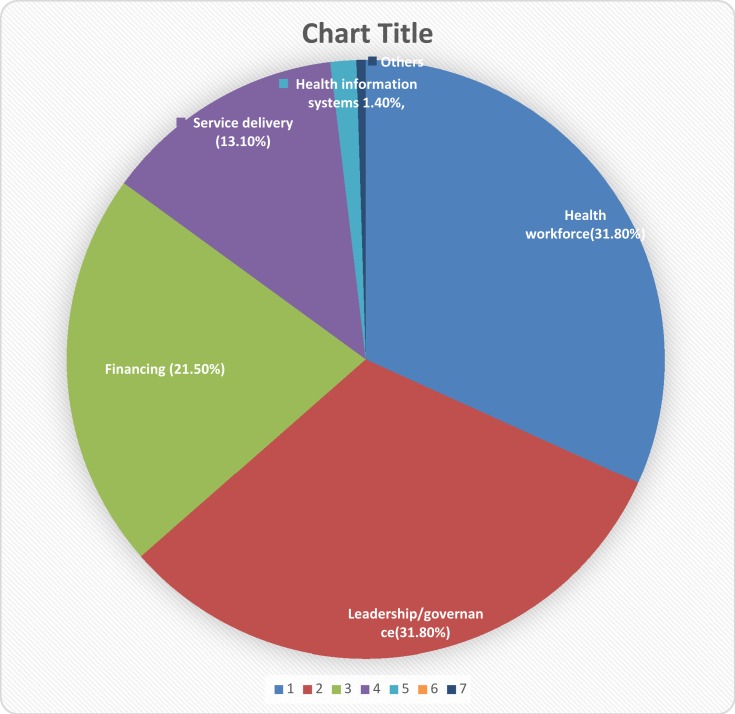
Solutions regrouped based on the six building blocks of the health system.

Further analysis of the suggested solutions based on the six WHO health system building blocks showed that the solutions proffered were majorly changes in leadership and governance (31.8%), healthcare workforce (31.8%) and healthcare financing (21.50%). These were followed by service delivery, healthcare information systems and access to medicines and technologies (Figure 2).

Solutions ranked first by the groups include training and capacity building of health workers (n =19, 29.69%), increased budgetary allocation to health (n =13, 20.31%), and advocacy for political support and commitment (n = 8, 12.50%). The rest is shown in Table 4.

**Table 4:** Key Solutions Ranked First In Africa

Variables	Frequency (N=64)	Proportion %
Training and capacity building of health workers	19	29.69
Increase budgetary allocation to health	13	20.31
Advocacy for political support and commitment	8	12.50
Health insurance for all	5	7.81
Improved leadership and management	5	7.81
Upgrade healthcare infrastructure	5	7.81
Strengthen partnership for health	4	6.25
Adequate accountability systems	2	3.13
Mental and social colonization	2	3.13
Improve quality of healthcare services	1	1.56

## Discussion

Healthcare systems in Africa are mostly in unworkable conditions with very poor health outcomes. There have been several studies that looked at the challenges of the healthcare system in Africa, and our major findings were to a large extent similar to previous studies.^10^,^11^,^22^ We found that the leading challenges in the healthcare sector as identified by the study participants were inadequate human resource for health, inadequate budgetary allocations to healthcare, and poor leadership and management in healthcare. These three problems accounted for over two-thirds of the perceived problems in the healthcare sector in Africa. When viewed from the perspective of the six WHO pillars of the healthcare system, the leading problems still clustered around leadership and governance, healthcare workforce and health service delivery and financing. Although this categorization may seem to overshadow the individual issues that make up these categories, it enables prioritization of the segments of the healthcare system most affected or requiring urgent attention. These findings are also similar to that of Roncarolo and colleagues, which found that the most frequently reported challenges in the health sector related to human resources (22%), leadership and governance (21%) and health service delivery (24%).^1^ The findings therefore suggest that addressing these three key challenges in the healthcare system may solve over two-third of the problems associated with the healthcare system in Africa.

At individual level, our study showed that inadequate human resource is the foremost leading challenge in the health sector in Africa. This is both overt and covert which reflects the complexity of this challenge. It is overt in the sense of inadequate number, mix and distribution resulting from inadequate output from training institutions; brain drain to Europe, America and Asia; and unfavourable governmental policies mitigating employment into the civil services. Covert reasons include poor availability of engaged healthcare workers to deliver services, resulting from healthcare worker strikes, attendance at private practice (rather than public hospitals), internal migration to big cities, and poor attitude to work.^23^–^25^ This is in line with the findings of WHO which showed that sub-Saharan Africa (SSA) faces the greatest challenges in shortage of human resource for health. Every component of the healthcare system depends on the people who manage and provide the services. For instance, healthcare provision depends very much on the people who provide the services to clients. Yet over the years, attention and support to the healthcare workforce has not received the priority it deserves.^25^ Little wonder, African countries are experiencing a disproportionate global disease burden of 24% with the least share of health workforce at only 3%.^26^ The same study showed that out of 57 countries globally classified as having critical healthcare workforce numbers, 36 of them are in sub-Saharan Africa; although some countries in Europe are beginning to have similar challenges^27^ These have implications at all level beginning with households especially those who are poor and those who live in rural areas who may have limited access to quality healthcare. This may be due to lack of qualified health personnel in health facilities which may lead to poor health outcomes and ultimately impede achievement of national and global health goals.^25^

Also, healthcare systems may not be able to respond to public health emergencies such as outbreak of diseases leading to increase mortality and morbidity in these countries. The major solution proffered to this key challenge centered on capacity building of healthcare workers, which may itself address this challenge to some extent. Other issues which influence the retention of healthcare workers also need to be investigated.

Inadequate financing of healthcare in Africa is another major challenge identified. This is in tandem with several other previous studies.^10^,^11^,^28^

For instance, Musango and colleagues in their studies found that in the majority of African countries, scarcity of funds for healthcare is a chronic problem with even the richest countries finding it increasingly difficult to keep up with rising healthcare costs, especially in the face of the ongoing economic downturn.^28^ Another study found that in about half of African countries, 40% or more of total healthcare expenditure is made up of out-of-pocket payments (OOPs), which are the most regressive way of funding healthcare. The average total healthcare expenditure (THE) in African countries stood at US$ 135 per capita in 2010, but only a small fraction of the US$ 3 150 spent on healthcare in an average high-income country.^29^ These poor healthcare financing indices are prevalent in Africa despite several declarations signed by African heads of state, which among others include the Abuja Declaration of 2001 on increasing government funding for health, and the 2012 Tunis Declaration on value for money, sustainability and accountability in the health sector.^28^

Although we identified low budgetary allocation to the healthcare sector and poverty as the key financial challenges faced by the healthcare systems, these are in contrast to those outlined by Roncarolo and colleagues which included increasing healthcare costs, financial unsustainability and lack of financial autonomy.^1^ The difference in findings may have resulted from the differing perspectives of the study participants. The implication of these findings lies in the fact that inadequate financing of healthcare by many governments leaves the burden for healthcare financing on households, usually through OOPs, which have been shown to be catastrophic to households, pushing some households into poverty. However, the main solutions proffered to the problem of inadequate financing of health are increasing budgetary allocation to healthcare and healthcare insurance for all. This solution may in itself be dependent on prevailing economic situations in these countries and other underlying factors which again account for the complexity of this challenge.

The third leading challenge identified in this study is poor leadership and management. This finding is in line with that noted by Dye and Garman, who stated that one of the biggest challenges in the coming decades will be the selection and development of leaders who are trained and prepared for leadership in increasingly complex healthcare systems.^30^ A study on leadership challenges in healthcare revealed that one of the underlying reasons for poor leadership stem from managers’ unfamiliarity with leadership techniques which in many cases is due to managers’ disbelief in the effectiveness and necessity of learning these techniques.^31^ Some other authors also documented similar challenges in two previous studies in Nigeria.^10^,^11^ Some of the leadership and management challenges mentioned by respondents include lack of political will, corruption in healthcare systems, poor resource management/inefficiencies and poor integration of healthcare programmes. These factors are similar to those noted in a previous study which outlined issues of poor governmental role in health policy and poor oversight of the whole health system^1^ The implications of these are that poor leadership could lead to increased healthcare costs, reduced efficiency and effectiveness, dissatisfaction among staff and ultimately resulting in lower patient satisfaction and poor health outcomes.^31^ Appropriate leadership, on the other hand, can create an organizational culture that is committed to quality, reducing conflicts, improving efficiency and productivity of teams, enhancing staff satisfaction, advancing healthcare system performance, and finally, meeting personal and organizational goals.^32^ Improved leadership and management have been proferred to address this challenge. There is an urgent need for capacity building on this subject which should be made mandatory beginning from healthcare training institutions and regular refresher course for all healthcare workers and finally should be a requirement for leadership in the healthcare sector.

In summary, the solutions proffered by the respondents which mostly addressed the issues outlined as the major challenges include training and capacity building for health workers, increase budgetary allocation to health, advocacy for political support and commitment, health insurance for all and improved leadership and management. Categorization of these solutions into the six WHO pillars of the healthcare system further showed that the major solutions outlined grouped into leadership and governance, healthcare workforce, and healthcare financing, accounting for over two-third of the suggested solutions. These imply that the solution to the key challenges in the healthcare system lies mainly in these three building blocks. These must be prioritized for action by policy makers and implementers if we desire to see better, healthier and more functional healthcare systems in Africa, translating into better healthcare outcomes and health indices.

## Strength And Limitations

The strength of this study is the variety of subjects drawn from across Africa and beyond, who are lettered and well versed in healthcare services and systems in Africa. The study also used self-administered questioner to avoid interviewer bias. The study has not been done anywhere before and thus provides a fresh perspective to the issues of healthcare systems in Africa, and results and recommendations are designed to improve healthcare systems in Africa.

The limitations include the sample size which was small and the fact that majority of the responders are epidemiologists at different career levels. There is therefore the need for a broader study involving not only epidemiologists, but also professionals and healthcare customers from other fields. However, the findings are very important and should both inform policy decisions in health, and development of strategies to improve healthcare outcomes in Africa.^33^

## Conclusion And Recommendation

Inadequate human resource and healthcare financing and poor leadership and management are the major challenges of healthcare systems in Africa. The major solutions outlined by the participants grouped into leadership and governance, healthcare workforce and healthcare financing. It is important for public health policy makers in Africa to adopt national guidelines for healthcare system development, based on the WHO framework with buy-in from healthcare workers and most importantly from governments across the region. Creative solutions are required and lack of resources may mean that public–private partnerships may need to be adopted with companies using corporate social responsibility schemes to provide a portion of profits to offset health insurance schemes for employees, their families and the communities in which they work. Legislation may be required to encourage large multinationals to participate in such schemes as the price for extracting raw resources from the African continent.
